# Comprehensive species set revealing the phylogeny and biogeography of Feliformia (Mammalia, Carnivora) based on mitochondrial DNA

**DOI:** 10.1371/journal.pone.0174902

**Published:** 2017-03-30

**Authors:** Yu Zhou, Si-Rui Wang, Jian-Zhang Ma

**Affiliations:** 1 College of Wildlife Resources, Northeast Forestry University, Harbin, China; 2 Feline Research Center of Chinese State Forestry Administration, Northeast Forestry University, Harbin, China; Senckenberg am Meer Deutsches Zentrum fur Marine Biodiversitatsforschung, GERMANY

## Abstract

Extant Feliformia species are one of the most diverse radiations of Carnivora (~123 species). Despite substantial recent interest in their conservation, diversification, and systematic study, no previous phylogeny contains a comprehensive species set, and no biogeography of this group is available. Here, we present a phylogenetic estimate for Feliformia with a comprehensive species set and establish a historical biogeography based on mitochondrial DNA. Both the Bayesian and maximum likelihood phylogeny for Feliformia are elucidated in our analyses and are strongly consistent with many groups recognized in previous studies. The mitochondrial phylogenetic relationships of Felidae were for the first time successfully reconstructed in our analyses with strong supported. When divergence times and dispersal/vicariance histories were compared with historical sea level changes, four dispersal and six vicariance events were identified. These vicariance events were closely related with global sea level changes. The transgression of sea into the lowland plains between Eurasia and Africa may have caused the vicariance in these regions. A fall in the sea level during late Miocene to Pliocene produced the Bering strait land bridge, which assisted the migration of American Feliformia ancestors from Asia to North America. In contrast with the ‘sweepstakes hypothesis’, our results suggest that the climate cooling during 30–27 Ma assisted Feliformia migration from the African mainland to Madagascar by creating a short-lived ice bridge across the Mozambique Channel. Lineages-through-time plots revealed a large increase in lineages since the Mid-Miocene. During the Mid-Miocene Climatic Optimum, the ecosystems and population of Feliformia rapidly expanded. Subsequent climate cooling catalyzed immigration, speciation, and the extinction of Feliformia.

## Introduction

Feliformia is a large suborder of Carnivora within the eutherian clade. The monophyletic origin of Feliformia is supported by molecular and morphological data [1–4]. According to Species 2000 and the databases ITIS Catalogue of Life [5] and Mammal Species of the World [6], Feliformia has six families (Felidae, Viverridae, Eupleridae, Nandiniidae, Herpestidae and Hyaenidae) with eight subfamilies (two in family Felidae, four in family Viverridae and two in family Eupleridae, while the families Herpestidae, Nandiniidae and Hyaenidae have no subfamilies), 54 genera and approximately 123 species. Due to pressures such as human hunting, habitat loss, lack of prey, and climate change, much of the extant Feliformia diversity is currently under extreme threat. According to The IUCN Red List of Threatened Species, Version 2016–2 [7], there are 12 endangered species: *Viverra megaspila*, *Prionailurus planiceps*, *Panthera uncia*, *Panthera tigris*, *Mungotictis decemlineata*, *Lynx pardinus*, *Leopardus jacobita*, *Galidictis grandidieri*, *Eupleres major*, *Cynogale bennettii*, *Chrotogale owstoni*, and *Catopuma badia*, and one species, *Cryptoprocta spelea*, has been classified as extinct.

Mitochondrial (mt) DNA is a useful marker system in phylogenetic analyses because of its maternal mode of inheritance and relative lack of recombination [8]. The mt genome holds a shorter expected coalescence time compared with nuclear loci; thus, there is a greater probability that the mt gene tree will accurately reflect the species tree [9]. Although phylogenetic analyses of mtDNA have proven problematic in some cases, such as the position of snakes among squamates [10] or the relationships of rodents among mammals [11], analyses of mtDNA are often congruent with those derived from nuclear genes when appropriate sampling of taxa and phylogenetic analyses are used [12,13]. In addition, comparatively few nuclear genes are widely used in phylogenetic analyses of Feliformia; for example, only Johnson et al. [14] used multiple nuclear genes to analyze the phylogenetic relationships of Felidae. Most other Feliformia taxa lack these genes. Thus, moderately sized mitochondrial genomes remain an attractive data resource for Feliformia phylogenetics.

A phylogenetic framework is critical to discover and conserve the diversity of extant Feliformia diversity. Previous taxonomic genetic sequencing and phylogenetic analysis have revealed many subgroups of Feliformia [3,14–24]. Several studies have suggested that increasing the number of sampled taxa enhances the accuracy of phylogenetic analyses (e.g., Rannala et al. [25]; Zwickl and Hillis [26]). However, Agnarsson et al. [24] evaluated the phylogenetic relationships of a comprehensive group of species of dogs, cats, and kin with only complete or partial mitochondrial Cytb gene sequences and obtained low support in many nodes of the Bayesian phylogeny tree, especially the nodes between the seven families within Feliformia. Thus, a large-scale and exhaustive species set is lacking for the phylogeny of extant Feliformia with multiple genes. A detailed historical biogeographic analysis of Feliformia also requires a comprehensive species data set to explore.

Here, we present an estimate of a comprehensive data set for the phylogeny and biogeography of Feliformia that includes 102 species (83% of the 123 known extant Feliformia species) from 52 of the 56 currently recognized genera (93%), as well as representatives from every currently delimited, extant family and subfamily base on the data matrix including up to 15,401 bp for each species from 37 complete mitochondrial genes. We also compared the divergence time with the lineages-through-time (LTT) plot and reconstructed the geographic range evolution of Feliformia with the global sea level and climate change information to discuss how global sea level change affected the evolution of Feliformia and how Feliformia responded to Cenozoic climatic change.

## Materials and methods

### Taxonomic reference

Our initial taxonomy was based on the 28 September 2015 update of the Species 2000 and ITIS Catalogue of Life database [5] and was similar to that of the Mammal Species of the World database [6] at the species and generic levels, with 6 families and 54 genera. The linsang lineage (Prionodon), which recent studies have suggested is closely related to Felidae [3,14,22], was still placed within Viverridae by Species 2000, the ITIS Catalogue of Life and Mammal Species of the World [5,6]. We agreed with the classification of Prionodon by Gaubert and Veron [22] and included it in the monogeneric family Prionodontidae in our taxonomy.

### Molecular data

In this study, we collected mitochondrial concatenated alignments consisting of 15,401 bp in 103 in-group species from GenBank and previous studies. These species included 83% of the currently recognized Feliformia species, representing 37 Felidae species in 15 genera (88% of 42 known extant species and 100% of 15 genera), 29 Viverridae species in 14 genera (82% of 35 species and 93% of 15 genera), 24 Herpestidae species in 12 genera (73% of 33 species and 86% of 14 genera), 6 Eupleridae species in 6 genera (75% of 8 species and 86% of 7 genera), and 4 Hyaenidae species in 3 genera (100% of 4 species and 100% of 3 genera), 2 belonging to the monogeneric family Prionodontidae and one (the only) species of the monogeneric family Nandiniidae. The concatenated alignment sequence matrix contained data from 103 species for cytb (100%, 402–1139 bp), 82 species for ND2 (79%, 696–1044 bp), and 45 species for the mitochondrial genome (exclude D-loop region) (56%, 15,401 bp). GenBank numbers are listed in S1 Table.

In this study, many taxa had large amounts of missing data (some >93%), and on average each species had 50% missing cells. However, two lines of evidence suggest that these missing data are not problematic. First, two genes (Cytb/ND2) were shared by the vast majority of taxa (100% and 76%, respectively), providing a “backbone” for the placement of most taxa based on overlapping sequence data. Our phylogeny results also suggest that this sampling design can be critically important for allowing the accurate placement of taxa with extensive missing data, as opposed to having nearly the same number species with only the Cytb gene sequence [24]. Second, several recent empirical studies have shown that the supermatrix approach (with extensive missing data in some taxa) yields generally well-supported large-scale trees that are in general highly congruent with both existing taxonomies and previous phylogenetic estimates (e.g., Driskell et al. [27]; McMahon and Sanderson [28]; Pyron et al. [29]; Thomson and Shaffer [30]; Wiens et al. [31]; Agnarsson et al. [24]).

### Sequence alignment, partition strategy and substitution model selection

Nucleotide sequences were aligned using Clustal X version 1.81 [32] with default parameters and manually optimized in MEGA version 5.0 [33]. Ambiguous alignments were removed using Gblocks version 0.91b [34] with the ‘with half’ option and default block parameters. We performed nucleotide diversity analyses based on the Cytb gene using MEGA software.

For the concatenated data set, 63 data blocks were defined (24 data blocks for RNA genes and 39 data blocks for protein genes). Each RNA gene represented a single block, and each protein gene was separated into three blocks based on codon positions. We defined ‘greedy’ partitioning strategies to compare partitioning strategies and selections of corresponding nucleotide substitution models under the Bayesian information criterion (BIC) using PartitionFinder version 1.1.1 [35]. The seven-partition scheme was chosen as the best-fitting partitioning strategy (S2 Table), and all seven partitions favored the GTR +I+G model.

### Phylogenetic analyses

Three Caniformia species (*Canis lupus*, *Vulpes lagopus* and *Martes flavigula*) were retrieved from GenBank and used as outgroups in phylogenetic analyses (for detailed information, see S1 Table). The concatenated data set was analyzed by maximum likelihood (ML) and Bayesian inference (BI) methods separately under the seven-partition scheme. Partitioned ML analyses were implemented using RAxML version 7.2.8 [36] with the GTR +I+G model assigned to each partition. A search that combined 200 separate ML searches was performed to identify the optimal tree, and branch support for each node was evaluated with 200 standard bootstrapping replicates (-f d -b 200 option) implemented in RAxML. The partitioned BI was conducted using MrBayes 3.2 [37] under the GTR +I+G model. All model parameters were unlinked. Four Markov chain Monte Carlo (MCMC) runs were performed with one cold chain and three heated chains (temperature set to 0.1) for 10 million generations and sampled every 1,000 generations. The first 25% of the generations were discarded, and topologies and posterior probabilities were estimated from the remaining generations.

### Estimates of divergence time

To further confirm the divergence time within Feliformia, another nine complete mitochondrial genomes of the following vertebrate species were retrieved from GenBank (All GenBank numbers see S1 Table) *Hippopotamus amphibius*, *Steno bredanensis*, *Balaenoptera physalus*, *Tapirus indicus*, *Bos grunniens*, *Equus caballus*, *Erignathus barbatus*, *Procyon lotor*, and *Tremarctos ornatus*. BEAST version 1.8.3 [38] software with a relaxed uncorrelated log-normal clock model [39] was used to date Feliformia. A Yule process was employed for each of the seven partitions, and fourteen log-normal prior constraints allowing ‘hard’ minimum and ‘soft’ maximum constraints were imposed on the tree to establish the divergence time (Table 1). For the ten in-group constraints with only minimum bounds, we set the maximum bound at 55 Ma based on arithmetic medians of Feliformia and Caniformia divergence [40]. The means and standard deviations of the log-normal distribution for each calibration point were selected to ensure 95% of the probability was within the minimum and maximum limits, and the means represented the arithmetic medians of the intervals. Analyses were performed under the same partitioned substitution model with BI phylogeny with 50 million generations while sampling every 1,000th tree, and the first 10% of the sampled trees were treated as burn-in. Burn-in and convergence of the chains were determined using the program Tracer v1.6 [41].

**Table 1:** Calibrations, divergence time and reconstructed ancestral areas of internal nodes within the phylogeny of Feliformia.

Node	Estimated Age (Ma)		Ancestral Area by Lagrange		Calibrations		
	mean	95% HPD	split	relative probability	lineage split/origin	age (Ma)	sources
1	43.12	37.87–49.29	BC|C	0.54			
2	38.89	32.95–41.68	BC|B	0.52			
3	35.00	31.26–39.66	C|BC	0.45			
4	29.51	26.12–33.44	C|C	0.6			
5	27.38	24.39–31.22	C|D	0.94			
6	18.67	17.47–20.61	C|C	0.83	Herpestidae*	>18	[42,43]
7	13.69	11.69–15.87	BC|C	0.55			
8	12.63	10.55–14.80	C|B	1			
9	19.99	15.72–24.48	D|D	1			
10	11.36	9.26–14.35	C|C	0.75	Crocuta–Hyaena*	>9.5	[44]
11	25.46	22.77–28.83	BC|B	0.66	Viverridae*	>23	[43,45]
12	8.43	6.87–10.56	C|C	1	Genetta*	>7.5	[46]
13	11.83	8.15–15.88	B|C	1			
14	32.27	28.23–36.96	B|B	0.76	Felidae-Prionodontidae	50–28	[22,47]
15	16.53	14.16–19.27	A|AB	0.8			
16	9.40	7.86–11.03	AC|A	0.51	Felis*	>4.2	[48,49]
17	9.36	7.68–11.18	C|C	1	Leptailurus serval*	>3.8	[48]
18	2.10	1.40–2.97	A|E	1			
19	7.89	6.29–9.58	E|A	1			
20	6.75	5.10–8.36	EF|E	0.95	Puma*	>1.8	[50]
21	11.21	9.26–13.63	AB|B	0.63	Panthera*	>3.8	[48]
22	8.14	6.58–9.96	A|AB	0.69	Tiger*	>1	[51,52]
23	5.87	4.62–7.34	A|A	0.68			
24	4.97	3.83–6.31	A|E	1			
25	11.68	7.33–16.82	B|B	1			
26	60.62	52.51–70.26			Felid-Canid*	65–45	[40]
27	54.29	50.94–60.05			Whale-Hippo*	60–51	[53]
28	33.72	32.32–35.64			Toothed whale-Baleen whale*	40–33	[53]
29	51.21	49.76–54.73			Horse-Tapir*	58–50	[54,55]

Shown are mean values and 95% highest posterior density (HPD) intervals of the ages and the subdivision/inheritance scenarios (‘split’) with the highest relative probability (RP) based on a dispersal-extinction-cladogenesis model in Lagrange. Asterisks (*) after calibrations indicate fossil calibration that we extract from the paleontological literature.

The LTT plot was constructed using the program Tracer v1.6 and the BEAST divergence time output files. Further, we compared interesting internal nodal ages (nodal age among Feliformia families, the crown age of each Feliformia family, obvious dispersal and vicariance nodal age) and the LTT plot with historical global sea level changes reported by Haq et al. [56] and historical global climate changes reported by Zachos et al. [57] to evaluate the vicariance and dispersal history of Feliformia and its response to historical global sea level changes and climate changes.

### Area delimitation and biogeographical reconstruction

We compiled the distribution data of Feliformia species from Species 2000 and the databases ITIS Catalogue of Life and the Mammal Species of the World [5,6]. The contemporary distribution ranges of Feliformia were divided into 6 areas based on their disjunctive distribution patterns and possible biogeographical barriers. Each Feliformia species was then assigned to its associated area according to its contemporary distribution range. The six areas were as follows: A, Europe-North Asia; B, South Asia; C, Africa; D, Madagascar; E, North America; and F, South America.

The ancestral area of each family of Feliformia was reconstructed using the dispersal-extinction-cladogenesis (DEC) model in Lagrange version 20130526 [58,59]. The DEC model specifies instantaneous transition rates among discrete distribution ranges along phylogenetic branches and uses the rates to access range inheritance scenarios at cladogenesis events [59]. The analysis was conducted based on the coded distribution range as defined above, and the chronogram of Feliformia species was estimated in BEAST. The maximum number of ancestral areas was limited to two, assuming that the dispersal of ancestors was similar to that of their extant descendants [60].

## Results

### Phylogenetic analyses

**Fig 1:**
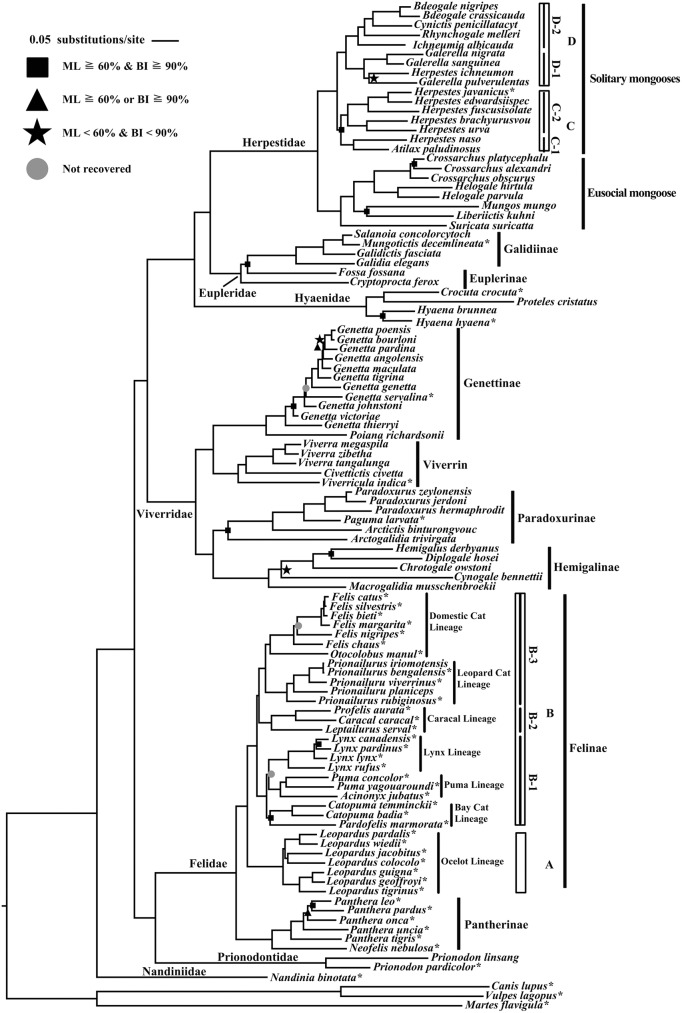
Topology of maximum likelihood analysis based on combined data on the mitochondrial genome. Nodes lacking support boxes exhibited >80% support from bootstrap proportions and >95% from Bayesian posterior probabilities. Asterisks (*) after the tip labels indicate individuals with mitochondrial genome sequence.

ML and BI analyses of the concatenated mitochondrial data set produced similar topologies. the ML tree is shown in Fig 1 and the BI tree is displayed in S1 Fig. This phylogeny was generally established, with 92% of nodes exhibiting ML bootstrap proportions (BP) greater than 0.6 and 92% of nodes with BI posterior probability (PP) greater than 0.9. Only five nodes exhibited weak support by ML (BP <0.6) and BI (PP<0.9) or yielded different topologies in the two analyses. Our analyses support a monophyletic origin of the families Nandiniidae, Prionodontidae, Herpestidae, Eupleridae, Hyaenidae, Viverridae and Felidae and also strongly supported the family-level relationships within Feliformia with a lineage of (Nandiniidae+ ((Felidae + Prionodontidae) + (Viverridae + (Hyaenidae + (Herpestidae + Eupleridae))))), consistent with most other studies of extant Carnivora and Feliformia phylogeny [1–3,21]. A majority of the phylogenetic relationships within each of these families remain unchanged compared to recent estimates [3,15,19]. However, we observed significant deviations from previous phylogenies and taxonomies, which we describe below, along with proposed solutions.

Within family Felidae, our results agree with other recent studies, indicating that the two monophyletic subfamilies Felinae and Pantherinae composed the family [14,61]. Six species of subfamily Pantherinae were represented in a progressive evolutionary model and were strongly supported in our analyses. Genus *Neofelis* and genus *Panthera* displayed a sister relationship, and *Uncia uncia* nested within *Panthera*, which is consistent with the results of Zhang and Zhang [61] and Johnson et al. [14]. Our analysis confirmed the phylogenetic relationship of *Panthera* species as (*P*. *tigris* + (*Uncia uncia* + (*P*. *onca*+(*P*. *leo* + *P*. *pardus*)))). The subfamily Felinae included two main clades: Clade A, Ocelot lineage (*Leopardus*); Clade B, other lineages. The monophyletic origin of each lineage and genus within Felinae was also strongly supported. Clade B consisted of three monophyletic subclades: subclade B-1, Bay Cat lineage, Puma lineage and Lynx lineage; subclade B-2, Caracal lineage; subclade B-3, Domestic Cat lineage and Leopard Cat lineage.

Within family Viverridae, our results are consistent with other recent studies supporting the corresponding relationship at the subfamily and generic levels [3,20,22]. The four monophyletic subfamilies displayed a strongly supported lineage ((Viverrin + Genettinae) + (Hemigalinae + Paradoxurinae)). The relationships within each subfamily were as follows: Viverrin, (*Viverricula*+(*Civettictis*+*Viverra*));Genettinae, (*Poiana*+*Genetta*); Paradoxurinae, (*Arctogalidia*+(*Arctictis*+(*Paguma*+*Paradoxurus*))); Hemigalinae, (*Macrogalidia*+(*Cynogale*+(*Chrotogale*+(*Diplogale*+*Hemigalus*)))). Furthermore, although *Prionodon* was classified within Viverridae by Wilson and Reeder [6] and Roskov et al. [5], our phylogenetic trees were consistent with Gaubert and Veron [22] in redefining Viverridae as excluding *Prionodon* and placing *Prionodon* in the monogeneric family Prionodontidae. The P-distances between *Prionodon* and each of the other families of Feliformia were 0.177–0.191 (S3 Table), larger than the average family P-distance (0.169) of the other 6 traditional families and consistent with the classification of Prionodon as the monogeneric family Prionodontidae.

Within the family Hyaenidae, Our ML and BI analyses were consistent with Koepfli et al. [21] in that *Crocuta* and *Proteles* first obtained a sister relationship (BP = 95, PP = 0.99) and then together constituted a monophyletic family with *Hyaena* (BP = 100, PP = 1.0).

Within the family Herpestidae, our results were similar to those of Patou et al. [19] that strongly supported two main sister groups (eusocial mongoose and solitary mongoose) within Herpestidae. The phylogenetic relationship of the eusocial mongoose clade indicated the following lineage: (*Suricata*+((*Mungos*+*Liberiictis*)+(*Helogale*+*Crossarchus*))). Within solitary mongooses, two main clades (Clade C and Clade D) were observed and displayed a sister relationship. Clade C included two subclades: the two African species *A*. *paludinosus* and *H*. *naso* as subclade C-1 and Asian mongooses (*Herpestes*) as subclade C-2. The African mongooses constituting Clade D also included two subclades with a sister relationship: subclade D-1 (*Galerella* + *H*. *ichneumon*) and subclade D-2 (*Ichneumia*+(*Rhynchogale*+(*Cynictis*+*Bdeogale*))).

Within the family Eupleridae, our phylogenetic trees supported the monophyly of subfamily Galidiinae as a lineage (((*Mungotictis*+*Salanoia*)+*Galidictis*)+*Galidia*) with the crown node, and all internal nodes had 100% support according to both ML and BI analyses. However, *Cryptoprocta ferox* and *Fossa fossana* of the subfamily Euplerinae did not fit into one lineage. Three clades (Galidiinae, *Cryptoprocta* and *Fossa*) constitute the Eupleridae according to our phylogeny analyses and display a relationship of ((Galidiinae+ *Fossa*)+*Cryptoprocta*) with strong support.

### Divergence times and ancestral areas

**Fig 2:**
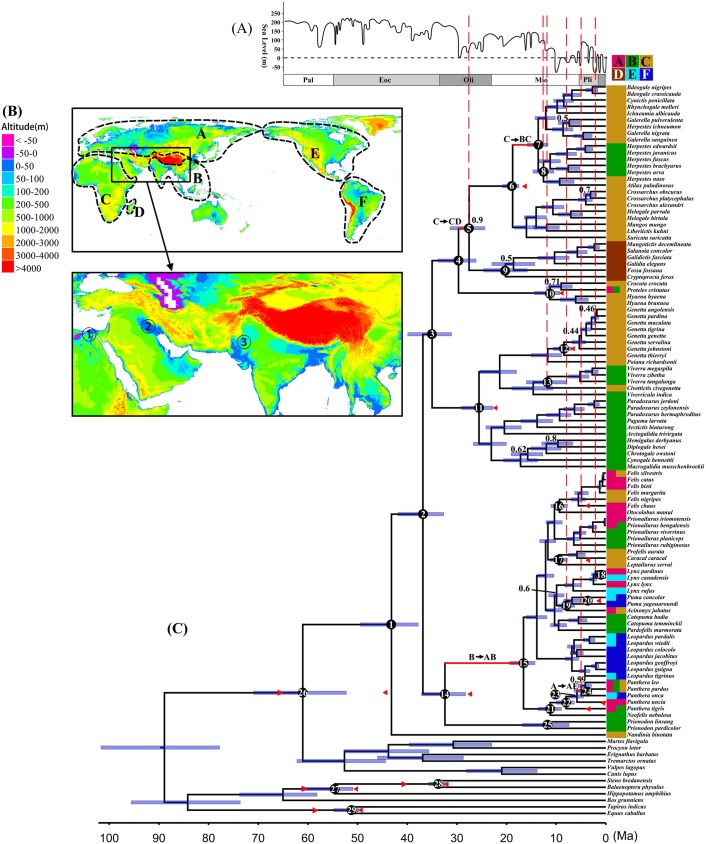
Chronogram and ancestral area reconstructions of Feliformia. (A) Major changes in global sea levels modified from Haq et al. [56]. Epoch abbreviations are as follows: Paleocene (Pal); Eocene (Eoc); Oligocene (Oli); Miocene (Mio); Pliocene (Pli). (B) Continental elevation map. Contemporary distribution ranges of Feliformia are divided into six clearly defined areas: A, Europe and North Asia; B, South Asia; C, Africa; D, Madagascar; E, North America; F, South America. The circles with numbers represent lowland plains: ① Nile Valley, ② Mesopotamian Plain, and ③ Indo-Gangetic Plain. (C) Chronogram and ancestral area reconstructions of Feliformia. Fourteen time constraints used in the molecular dating are shown as red triangles. Red branch with black arrows indicates dispersal events. Blue horizontal bars represent 95% HPD intervals. At the top right, a color-coded square represents the six main regions corresponding to continental elevation Map (A). Less than 0.95 branch support values are indicated beside the nodes. The red dash lines on black circles represent vicariance events.

The topology of the chronogram shown in Fig 2 is nearly identical to that of the phylogenetic trees. The mean and 95% highest posterior density (HPD) intervals of our fourteen constrained nodes were all within our constraints. The crown age of suborder Feliformia is circa 43 Ma. The age of the internal nodes among families of Feliformia can be traced to the late Eocene and the middle Oligocene (Fig 2 and Table 1). The definitions of the numbers of the nodes and abbreviations of area delimitations (A–F) within Table1 are provided in Fig 2. The detail divergence information of internal nodes at the family levels, the ancestor nodes of each family and other obvious dispersal and vicariance nodes are shown in Table 1.

**Fig 3:**
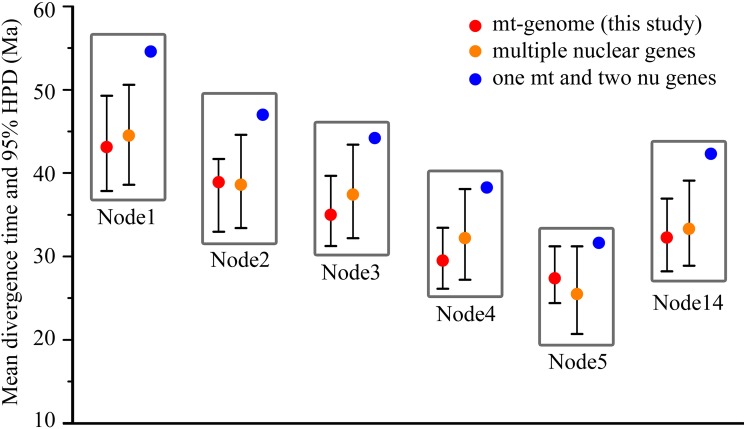
Comparison of divergence-time estimates among the seven Feliformia families across three studies. The circle within boxes represents the mean of the posterior estimate, and the whiskers mark the upper and lower 95% highest posterior density of the age estimates. The comparison shows that our new time estimates are largely congruent with previous results based on multiple nuclear genes but considerably younger than those estimated by one mitochondrial Cytb gene and two nuclear genes. Nodes in this figure correspond to Table 1.

When comparing our new divergence time estimates with the fossil records, all means and 95% HPD intervals of divergence times at the constraint nodes obtained by BEAST are within the constraint intervals we set. Divergence times estimated among the seven Feliformia families in the present study are close to those estimated with multiple nuclear gene sequences (Eizirik et al. [62]) but considerably younger than those from one mitochondrial Cytb gene and two nuclear genes (Gaubert and Cordeiro-Estrela [3]) (Fig 3). Using multiple nuclear loci has become the standard method of resolving species relationships in difficult biological scenarios [63], and more nuclear loci used during the phylogenetic analyses lead to the phylogenetic relationship being closer to saturation [64]. Our divergence time estimated results are close to those of Eizirik et al. [62], indicating that the mt-genome is appropriate for Feliformia phylogeny analyses.

The ancestral range of subdivision/inheritance scenarios for each node of interest was initially analyzed by the Lagrange program (Table 1). Four dispersal and seven vicariance events (RP of relative nodes >0.6) are shown in Fig 2 (indicated by red branch and arrows) and were inferred as follows: (1) a dispersal event occurred from Africa (C) to Madagascar (D) between nodes 4 and 5, followed by a vicariance event between the two areas on node 5; (2) a dispersal event occurred from Africa to South Asia (B) between nodes 6 and 7, followed by a vicariance event between the two areas on node 8; (3) a vicariance event occurred between Africa and South Asia on node 13; (4) a dispersal event occurred from South Asia to Europe-North Asia (A) between node 14 and 15; (5) three vicariance events occurred between Europe-North Asia and North America (E) on node 18, 19 and 24, respectively; (6) a dispersal event occurred from Europe-North Asia to North America between nodes 23 and 24, followed by a vicariance event between the two areas on node 24. According to the Lagrange analysis, the ancestral ranges of the 7 families were determined as follows: Nandiniidae, Africa; Felidae, South Asia; Prionodontidae, South Asia; Herpestidae, Africa; Eupleridae, Madagascar; Hyaenidae, Africa; Viverridae, Eurasia.

Although some recent studies have estimated different sea level change histories using various methods (e.g., Haq et al. [56]; Haq and AI-Quahtani [65]; Miller et al. [66]; Miller et al. [67]), many of these sea level change results have the same variation trends as our dispersal event periods and vicariance event times. Thus, we only show the global sea level change history by Haq et al. [56] as representative in Fig 2. The timing of the vicariance nodes 5, 8, 13, 18, 19 and 24 was closely related to historical global sea level changes (Fig 2). The mean times of vicariance nodes 8 and 13 are located at the relative crest value during Middle Miocene sea level changes, respectively. The mean times of vicariance nodes 18, 19 and 24 are located at or near the relative trough during Late Miocene to Pliocene sea level changes, respectively. The 95% PHD intervals of these vicariance nodes also crossed at least one sea level rise or fall history. All four dispersal events have at least one rapid sea level fall during the dispersal branch times. The vicariance event between African mainland and Madagascar on node 5 was at a relatively low sea level period in the Middle Oligocene, 27.4 (24.39–31.22) Ma.

### Lineages through time

**Fig 4:**
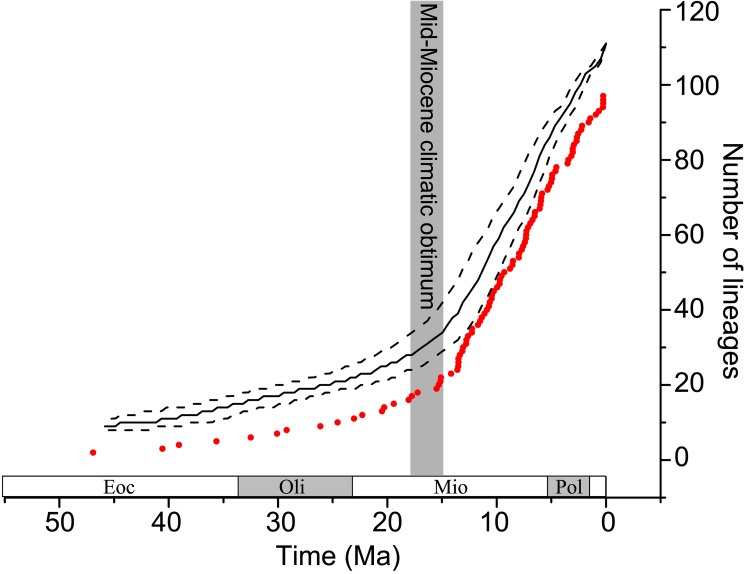
The LTT plot of Feliformia. An expected lineages-through-time plot was analyzed based on the BEAST MCC tree with median (black solid line) and 95% CI (black dotted lines) numbers of lineages through time shown. The observed lineage-through-time plot (red filled circles) was statistically based on the time tree (Fig 2).

The expected LTT plot of Feliformia species was constructed by the Tracer program using the output tree files from the BEAST divergence time analyses. Expected and observed LTT plots hold a similar increasing tendency, and the lineage number of the expected LTT plot was higher than the observed LTT plot (Fig 4) because the expected LTT plots were calculated under the birth–death process [68] and because there are predicted extinction lineages in the LTT plot. The number of lineages slowly increased before the middle Miocene and sharply thereafter. Comparison of the LTT plots with historical changes in global temperature [57] revealed a strong linear increase in the number of Feliformia lineages with gradual cooling of the climate after the Mid-Miocene Climatic Optimum.

## Discussion

### Phylogeny

**Fig 5:**
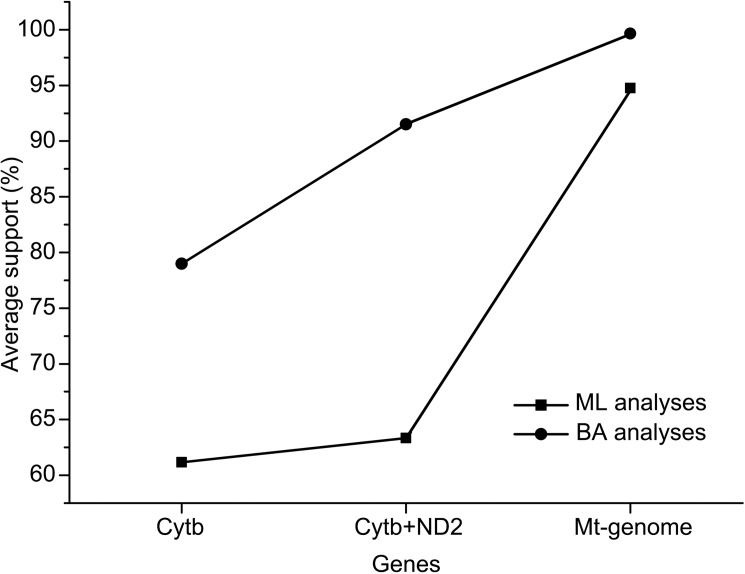
The effect of three data subsets (Cytb gene, Cytb and ND2 genes, mt-genome) on resolving support rates among the 7 families of Feliformia. Each data point represents the mean of support values estimated by BI and ML analyses.

Several studies have suggested that increasing the number of sampled taxa enhances the accuracy of phylogenetic analyses (e.g., Rannala et al., [25]; Zwickl and Hillis, [26]); thus, increased taxonomic sampling was performed to obtain the present data matrix. We included 86% of genera within Feliformia to successfully reconstruct the phylogenetic trees. The relationships between and within 7 monophyletic families were strongly supported by the phylogenetic analyses. Our analysis of three data subsets (Cytb gene, Cytb and ND2 genes, complete mt-genome) indicates a progressive increase in the average bootstrap support value for the six nodes among the 7 monophyletic families of Feliformia when an increasing number of genes are analyzed (Fig 5) and the complete mitochondrial genome subset, for which each Feliformia family has at least one species holding the complete mitochondrial genome sequence, shows the greatest performance according to the phylogenetic analyses.

For Herpestidae, Hyaenidae and Viverridae, phylogenetic relationships within each family were strongly supported in our analyses, and the results were consistent with previous studies [3,19,20,22]. For taxonomies within the genus *Herpestes*, our analyses indicated that *H*. *ichneumon* should be included within the genus *Galerella* and that *H*. *naso* is a close relative of *Atilax paludinosus*.

The relationships between all species of Euplerinae and some species of Galidiinae have been analyzed by Poux et al. [69] based on nuclear genes. Poux et al. also confirmed three major divisions within Eupleridae, including the Galidiinae clade, *Cryptoprocta ferox* clade, and the *Fossa* and *Eupleres* clades. Our analyses revealed similar nucleotide diversity between the three major clades (*Galidiinae*, *Cryptoprocta ferox* and *Fossa fossana*) with P-distances of 0.143 for Galidiinae vs. *C*. *ferox*, 0.130 for Galidiinae vs. *F*. *fossana*, and 0.142 for *C*. *ferox* vs. *F*. *fossana*. These findings are consistent with the three major divisions within the family Eupleridae.

Prionodon exhibited the same phylogenetic relationship observed in previous analyses of Felidae’s closest relative but did not belong to Viverridae [3,14,22]. Our phylogeny and P-distance were consistent with Gaubert and Veron’s [22] classification of Prionodon as the monogeneric family Prionodontidae.

Our mitochondrial phylogenetic analyses successfully reconstruct the phylogenetic tree for Felidae. The phylogenetic relationships between eight Felidae lineages were well resolved with the exception of the relationships between the Lynx lineage, Puma Lineage and Bay Cat lineage. The subfamily Felinae consisted of two main clades with four subclades. Our analyses clearly showed that the phylogenetic relationships between subfamily Pantherinae species were strongly represented by (*Panthera tigris* + (*Uncia uncia* + (*P*. *onca*+(*P*. *leo* + *P*. *pardus*)))). The taxonomies of *Uncia uncia* included a part of *Panthera*, which were also consistent with many previous mitochondrial phylogeny studies [14,24,61], and we agree with Yu et al. [70] that *Unica* is congeneric with *Panthera*.

### Historical biogeography

Our ancestral area reconstructions show that Feliformia were distributed in South Asia and Africa before the Middle Miocene and then dispersed to America several times since the Late Miocene. Three lowland plains (the Mesopotamian Plain, the Nile Valley and the Indo-Gangetic Plain) border the three main Feliformia dispersal realms: Europe-North Asia, South Asia, and Africa. Since the Eocene, global sea levels have changed greatly and repeatedly risen by more than 100 meters [56]. The separation of these dispersal realms by drastically rising sea levels to cover the lowland plains may have contributed to vicariances and speciation events during Feliformia evolutionary history. For nodes 8 and 13, two vicariance events occurred between South Asia and Africa. The mean ages of nodes 8 and 13 are at a sea level increase period, and the 95% PHD intervals of these vicariance nodes also crossed at least one sea level rise process. Before node 8, a dispersal event from Africa to South Asia between nodes 6 and 7 occurred during two of the lowest sea levels, which were approximately 50 m higher than present, at 17–15 Ma throughout the Late Oligocene to Middle Miocene. The Eurasian and African Feliformia could spread through the lowland plains during sea level falls and separate from each other by marine transgression into the lowland plains when sea levels rise. Thus, the most reasonable vicariances of these nodes were a separation between South Asia and Africa by the transgression of the lowland plain during a period of sea level rise. Nodes 18, 19 and 24 display three vicariance events between North America and Europe-North Asia, which were separated by the Bering Strait. Before node 24, there was a rapid sea level fall during the dispersal event between nodes 23–24, followed by a vicariance with a sea level rise. The sea level fall could have facilitated neritic region exposure to provide Feliformia with the opportunity to disperse across the Bering Strait. Thus, we think that the Bering Strait provided a land bridge during rapid decreases in sea level to facilitate the dispersion of American Feliformia ancestors from North Asia to North America, followed by a vicariance between the two areas when the sea level rose.

Although the tectonic plate movement of Madagascar relative to Africa began in the middle Jurassic and ended in the Early Cretaceous [71], several studies have determined that the terrestrial vertebrates on the island of Madagascar, including amphibians and mammals, originated on mainland Africa and migrated to Madagascar between the middle Paleocene and Oligocene [69,72,73]. No evidence supports a terrestrial bridge across the Mozambique Channel. The ‘sweepstakes hypothesis,’ which states that ancestors of Madagascar’s present-day mammalian stock rafted there from Africa, has been recognized as a valid colonization method [72,74–76]. Our divergence time indicated that mainland African Herpestidae ancestors were separated from their Madagascar-based Eupleridae ancestors at circa 27 Ma (95% HPD: 24.39–31.22 Ma) at a relatively lower sea level period that occurred during Oligocene global sea level changes (Fig 2). If the ‘sweepstakes hypothesis’ is valid, the relatively low sea level could have reduced the distance from mainland Africa to the island of Madagascar, thereby improving the success rate of Eupleridae ancestor over-water dispersal from mainland African to Madagascar. Moreover, we contrasted the dispersal time (30–27 Ma) of the Madagascar Eupleridae ancestor with global climate change history and found that the global climate was at an extraordinarily low temperature during this time, as estimated by many benthic foraminiferal oxygen isotopes (δ^18^O) studies [57,77–79]. The continental ice sheets, perhaps even sea ice, were present at such a low global temperature climate [80]. Thus, there was likely a short-lived bridge of sea ice across the Mozambique Channel that connected Madagascar and Africa; however, we did not find any specific records or references to this. If the Madagascar-Africa sea ice existed, the migration of the Madagascar Feliformia ancestor could shorten the migration time from 25–30 days according to the ‘sweepstakes hypothesis’ [81–83] to several days, making the migration more feasible. Our results suggest that the cooling climate during 30–27 Ma may have created a short-lived ice bridge connecting Madagascar and the Africa mainland, which assisted the migration of Madagascar Feliformia out of the African mainland.

### The response of Feliformia to Cenozoic climatic change

Both rapid and gradual climatic changes directly influenced the evolution and ecology of mammalian species and communities throughout the Cenozoic [84]. One of the most intriguing climatic events of the Neogene was the global warming event in the Middle Miocene (~15–18 Ma) known as the Mid-Miocene Climatic Optimum [85], after which Earth's climate gradually cooled while ice volumes increased globally [86,87]. Climatic changes during the Middle Miocene were associated with a sharp increase in the evolutionary turnover of both terrestrial and marine biota [85]. Our LTT plots also suggest that the climate since the Middle Miocene has been conducive to Feliformia diversification and speciation. The significant increase in lineages began at the Mid-Miocene climatic optimum and continued until the Pliocene. Similar increases also occurred in groupers [88]. Our hypothesis suggests that ecosystem and population expansion of Feliformia occurred rapidly during the Mid-Miocene Climatic Optimum. Subsequent climate cooling until the Pliocene, which could have directly catalyzed immigration, speciation, and the extinction of Feliformia, was a significant factor contributing to the Neogene evolutionary history of Feliformia species.

## Supporting information

S1 Fig The Bayesian inference tree. Nodes lacking support have support >95%.
(TIF)

S1 Table GenBank accession numbers for the 103 sampled Feliformia taxa and outgroup species. (DOCX)

S2 Table Substitution models selected in PartitionFinder using the Bayesian information criterion (BIC). (DOCX)

S3 Table Uncorrected p-distance between Cytb fragments from 7 families within Feliformia. (DOCX)
