# Supplementary figures and images for: Comprehensive species set revealing the phylogeny and biogeography of Feliformia (Mammalia, Carnivora) based on mitochondrial DNA
Source: PLoS One. 2017 Mar 30;12(3):e0174902. doi: 10.1371/journal.pone.0174902 (PMC5373635; doi:10.1371/journal.pone.0174902)

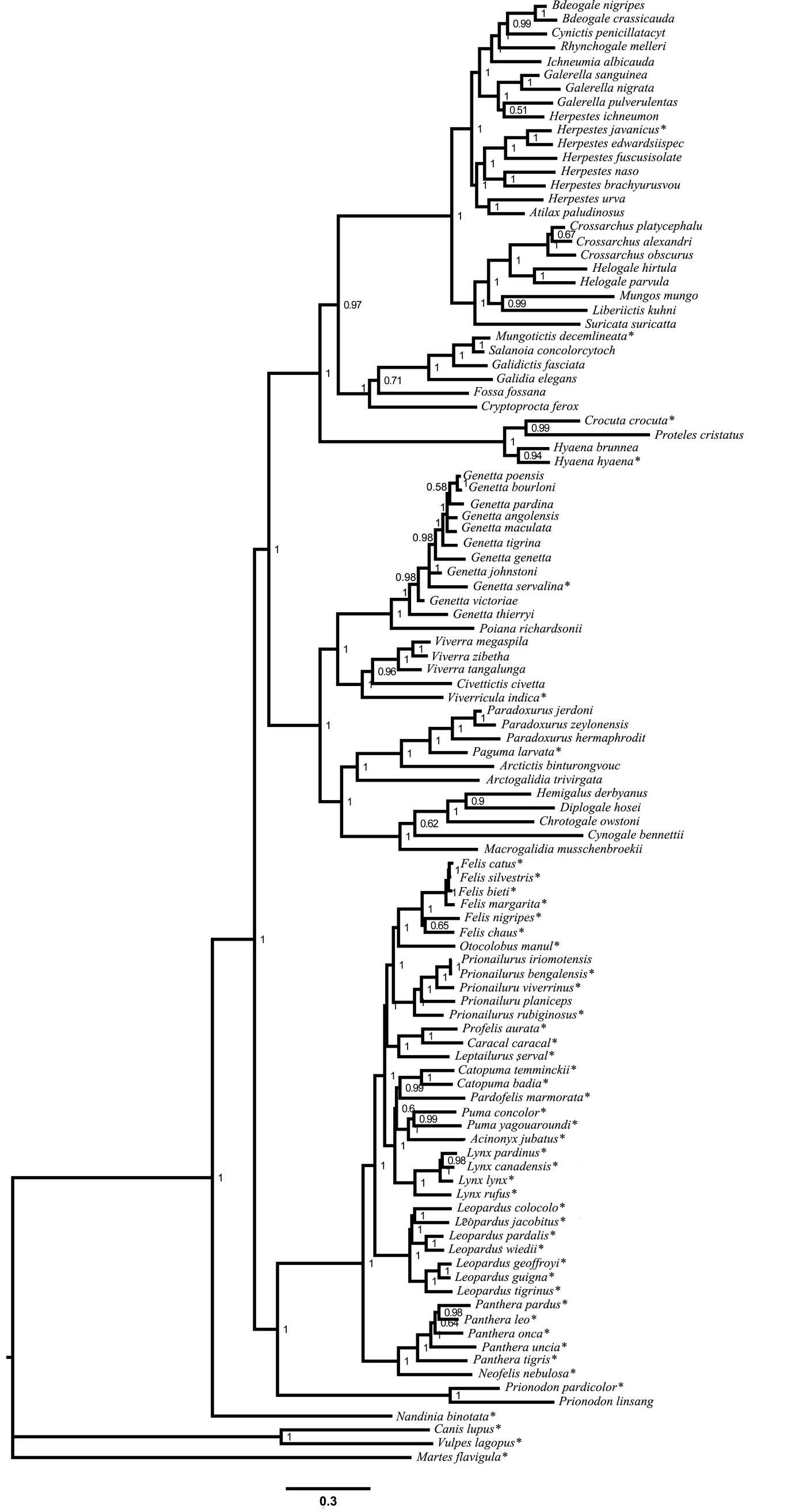

Supplement: S1 Fig — Nodes lacking support have support >95%. (TIF) [file pone.0174902.s001.tif]
