# Supplementary material for: Comprehensive species set revealing the phylogeny and biogeography of Feliformia (Mammalia, Carnivora) based on mitochondrial DNA
Source: PLoS One. 2017 Mar 30;12(3):e0174902. doi: 10.1371/journal.pone.0174902 (PMC5373635; doi:10.1371/journal.pone.0174902)
Supplement: S1 Table — (DOCX) [file pone.0174902.s002.docx]

**S1 Table. GenBank accession numbers references of 103 sampled Feliformia taxa and outgroup species.**

| Taxon | Ref. | Mt-genome | Cytb | ND1 | ND2 | ND4 | ND5 | 16S | ATP8 | Sequence lenth (bp) | Sequence data PCT (%) |
| --- | --- | --- | --- | --- | --- | --- | --- | --- | --- | --- | --- |
| *Acinonyx jubatus* | [[1](#_ENREF_1)] | AY463959 | --- | --- | --- | --- | --- | --- | --- | 15397 | 100 |
| *Caracal caracal* | [[2](#_ENREF_2)] | KP202272 | --- | --- | --- | --- | --- | --- | --- | 15327 | 100 |
| *Catopuma temminckii* | [[3](#_ENREF_3)] | KP271500 | --- | --- | --- | --- | --- | --- | --- | 15322 | 99 |
| *[Catopuma badia](https://www.ncbi.nlm.nih.gov/nuccore/NC_028300.1)* |  | KP202256 | --- | --- | --- | --- | --- | --- | --- | 15327 | 100 |
| *Felis bieti* | [[2](#_ENREF_2)] | KP202273 | --- | --- | --- | --- | --- | --- | --- | 15328 | 100 |
| *Felis catus* | [[4](#_ENREF_4)] | FCU20753 | --- | --- | --- | --- | --- | --- | --- | 15399 | 100 |
| *Felis chaus* | [[2](#_ENREF_2)] | KP202274 | --- | --- | --- | --- | --- | --- | --- | 15327 | 100 |
| *Felis margarita* | [[5](#_ENREF_5)] | KR132580 | --- | --- | --- | --- | --- | --- | --- | 15400 | 100 |
| [*Felis nigripes*](http://www.catalogueoflife.org/col/details/species/id/10ce4d605a3b3dc0933b2debdb40fdc0/source/tree) | [[2](#_ENREF_2)] | KP202277 | --- | --- | --- | --- | --- | --- | --- | 15329 | 100 |
| *Felis silvestris* | [[6](#_ENREF_6), [7](#_ENREF_7)] | KP202278 | --- | --- | --- | --- | --- | --- | --- | 15326 | 100 |
| *Leopardus colocolo* | [[2](#_ENREF_2)] | KP202282 | --- | --- | --- | --- | --- | --- | --- | 15325 | 100 |
| *Leopardus geoffroyi* | [[2](#_ENREF_2)] | KP202292 | --- | --- | --- | --- | --- | --- | --- | 15328 | 100 |
| *Leopardus guigna* | [[2](#_ENREF_2)] | KP202293 | --- | --- | --- | --- | --- | --- | --- | 15328 | 100 |
| *Leopardus jacobitus* | [[2](#_ENREF_2)] | KP202294 | --- | --- | --- | --- | --- | --- | --- | 15329 | 100 |
| *Leopardus pardalis* | [[2](#_ENREF_2)] | KP202284 | --- | --- | --- | --- | --- | --- | --- | 15328 | 100 |
| *Leopardus tigrinus* | [[2](#_ENREF_2)] | KP202287 | --- | --- | --- | --- | --- | --- | --- | 15328 | 100 |
| *Leopardus wiedii* | [[2](#_ENREF_2)] | KP202289 | --- | --- | --- | --- | --- | --- | --- | 15327 | 100 |
| *Leptailurus serval* | [[2](#_ENREF_2)] | KP202286 | --- | --- | --- | --- | --- | --- | --- | 15326 | 100 |
| *Lynx rufus* |  | GQ979707 | --- | --- | --- | --- | --- | --- | --- | 15252 | 99 |
| *Lynx lynx* | [[5](#_ENREF_5)] | KM982549 | --- | --- | --- | --- | --- | --- | --- | 15323 | 100 |
| *Lynx pardinus* | [[5](#_ENREF_5)] | KR132583 | --- | --- | --- | --- | --- | --- | --- | 15314 | 99 |
| *Lynx canadensis* | [[8](#_ENREF_8)] | KP202281 | --- | --- | --- | --- | --- | --- | --- | 15324 | 100 |
| *Pardofelis marmorata* | [[2](#_ENREF_2)] | KT288227 | --- | --- | --- | --- | --- | --- | --- | 15327 | 100 |
| *Prionailurus iriomotensis* | [[9](#_ENREF_9)] | --- | AB210228 | --- | --- | --- | --- | --- | --- | 1140 | 7 |
| *Prionailurus planiceps* | [[8](#_ENREF_8)] | --- | FJ594958 | # | # | # | # | # | # | 3916 | 25 |
| *Prionailurus bengalensis* |  | HM185183 | --- | --- | --- | --- | --- | --- | --- | 15397 | 100 |
| *Prionailurus viverrinus* | [[5](#_ENREF_5)] | KR132596 | --- | --- | --- | --- | --- | --- | --- | 15056 | 98 |
| *Prionailurus rubiginosus* | [[5](#_ENREF_5)] | KR132593 | --- | --- | --- | --- | --- | --- | --- | 15056 | 98 |
| *Profelis aurata* | [[2](#_ENREF_2)] | KP202255 | --- | --- | --- | --- | --- | --- | --- | 15325 | 100 |
| *Puma concolor* |  | JN999997 | --- | --- | --- | --- | --- | --- | --- | 15252 | 99 |
| *[Puma yagouaroundi](https://www.ncbi.nlm.nih.gov/nuccore/KP202279.1)* | [[2](#_ENREF_2)] | KP202279 | --- | --- | --- | --- | --- | --- | --- | 15329 | 100 |
| *Otocolobus manul* | [[5](#_ENREF_5)] | KR132585 | --- | --- | --- | --- | --- | --- | --- | 12926 | 84 |
| *Neofelis nebulosa* | [[10](#_ENREF_10)] | DQ257669 | --- | --- | --- | --- | --- | --- | --- | 15322 | 99 |
| *Panthera pardus* | [[11](#_ENREF_11)] | EF551002 | --- | --- | --- | --- | --- | --- | --- | 15397 | 100 |
| *Panthera tigris* | [[12](#_ENREF_12)] | KF297576 | --- | --- | --- | --- | --- | --- | --- | 15401 | 100 |
| *Panthera onca* |  | KF483864 | --- | --- | --- | --- | --- | --- | --- | 15400 | 100 |
| *Panthera leo* |  | KF776494 | --- | --- | --- | --- | --- | --- | --- | 15400 | 100 |
| *Uncia uncia* | [[13](#_ENREF_13)] | EF551004 | --- | --- | --- | --- | --- | --- | --- | 15325 | 100 |
| *Arctictis binturong* | [[14](#_ENREF_14), [15](#_ENREF_15)] | --- | KJ852005 | --- | DQ683980 | --- | --- | --- | --- | 2181 | 14 |
| *Arctogalidia trivirgata* | [[15](#_ENREF_15), [16](#_ENREF_16)] | --- | AF125140 | --- | DQ683982 | --- | --- | --- | --- | 2182 | 14 |
| *Macrogalidia musschenbroekii* | [[17](#_ENREF_17)] | --- | JQ219116 | --- | JQ219114 | --- | --- | --- | --- | 1047 | 7 |
| *Paguma larvata* | [[15](#_ENREF_15), [18](#_ENREF_18)] | KT191130 | --- | --- | --- | --- | --- | --- | --- | 15319 | 99 |
| *Paradoxurus hermaphroditus* | [[7](#_ENREF_7), [19](#_ENREF_19)] | --- | AF511056 | --- | AY170056 | --- | --- | --- | --- | 2184 | 14 |
| *Paradoxurus jerdoni* | [[15](#_ENREF_15)] | --- | DQ683994 | --- | DQ683989 | --- | --- | --- | --- | 1202 | 8 |
| *Paradoxurus zeylonensis* | [[14](#_ENREF_14)] | --- | KJ852026 | --- | --- | --- | --- | --- | --- | 1020 | 7 |
| *Chrotogale owstoni* | [[15](#_ENREF_15), [16](#_ENREF_16)] | --- | AF125144 | --- | DQ683984 | --- | --- | --- | --- | 2162 | 14 |
| *Cynogale bennettii* | [[15](#_ENREF_15)] | --- | DQ683992 | --- | DQ683992 | --- | --- | --- | --- | 2160 | 14 |
| *Diplogale hosei* | [[17](#_ENREF_17)] | --- | JQ219112 | --- | JQ219110 | --- | --- | --- | --- | 1166 | 8 |
| *Hemigalus derbyanus* | [[7](#_ENREF_7)] | --- | AY170109 | --- | AY170052 | --- | --- | --- | --- | 2184 | 14 |
| *Prionodon linsang* | [[16](#_ENREF_16)] | --- | AF125141 | --- | --- | --- | --- | --- | --- | 1123 | 7 |
| *Prionodon pardicolor* | [[20](#_ENREF_20)] | KJ636050 | --- | --- | --- | --- | --- | --- | --- | 15391 | 100 |
| *Civettictis civegenetta* | [[7](#_ENREF_7)] | --- | AY170105 | --- | AY170048 | --- | --- | --- | --- | 1140 | 7 |
| *Genetta angolensis* | [[21](#_ENREF_21)] | --- | DQ395207 | --- | --- | --- | --- | --- | --- | 1125 | 7 |
| *Genetta bourloni* | [[22](#_ENREF_22)] | --- | AY241898 | --- | --- | --- | --- | --- | --- | 1000 | 6 |
| *Genetta genetta* | [[15](#_ENREF_15), [23](#_ENREF_23)] | --- | AY397697 | --- | DQ683985 | --- | --- | --- | --- | 2184 | 14 |
| *Genetta johnstoni* | [[24](#_ENREF_24)] | --- | DQ267557 | --- |  | --- | --- | --- | --- | 1140 | 7 |
| *Genetta maculata* | [[15](#_ENREF_15), [23](#_ENREF_23)] | --- | AY397721 | --- | DQ683986 | --- | --- | --- | --- | 1140 | 7 |
| *Genetta pardina* | [[23](#_ENREF_23)] | --- | AY397707 | --- | --- | --- | --- | --- | --- | 1140 | 7 |
| *Genetta poensis* | [[22](#_ENREF_22)] | --- | AY241897 | --- | --- | --- | --- | --- | --- | 1000 | 6 |
| *Genetta servalina* | [[25](#_ENREF_25)] | KJ624980 | --- | --- | --- | --- | --- | --- | --- | 15392 | 100 |
| *Genetta thierryi* | [[22](#_ENREF_22)] | --- | AY241893 | --- | --- | --- | --- | --- | --- | 1140 | 7 |
| *Genetta tigrina* | [[23](#_ENREF_23)] | --- | AY397705 | --- | --- | --- | --- | --- | --- | 1140 | 7 |
| *Genetta victoriae* | [[22](#_ENREF_22)] | --- | AY241894 | --- | --- | --- | --- | --- | --- | 1000 | 6 |
| *Poiana richardsonii* | [[22](#_ENREF_22)] | --- | AY241891 | --- | --- | --- | --- | --- | --- | 1140 | 7 |
| *Viverra megaspila* | [[19](#_ENREF_19)] | --- | AF511046 | --- | --- | --- | --- | --- | --- | 1140 | 7 |
| *Viverra zibetha* | [[16](#_ENREF_16)] | --- | AF125146 | --- | --- | --- | --- | --- | --- | 1140 | 7 |
| *Viverra tangalunga* | [[7](#_ENREF_7), [19](#_ENREF_19)] | --- | AF511045 | --- | AY170055 | --- | --- | --- | --- | 2184 | 14 |
| *Viverricula indica* | [[26](#_ENREF_26)] | KM276554 | --- | --- | --- | --- | --- | --- | --- | 15388 | 100 |
| *Cryptoprocta ferox* | [[7](#_ENREF_7), [16](#_ENREF_16)] | --- | AF125139 | --- | AY170036 | --- | --- | --- | --- | 2184 | 14 |
| *Fossa fossana* | [[7](#_ENREF_7)] | --- | AY170097 | --- | AY170037 | --- | --- | --- | --- | 1202 | 8 |
| *Galidia elegans* | [[7](#_ENREF_7), [27](#_ENREF_27)] | --- | AY170099 | --- | AY750648 | --- | --- | --- | --- | 1202 | 8 |
| *Galidictis fasciata* | [[7](#_ENREF_7)] | --- | AY170100 | --- | AY170040 | --- | --- | --- | --- | 2184 | 14 |
| *Mungotictis decemlineata* | [[28](#_ENREF_28)] | JN378116 | --- | --- | --- | --- | --- | --- | --- | 1140 | 7 |
| *Salanoia concolor* | [[7](#_ENREF_7)] | --- | AY187007 | --- | --- | --- | --- | --- | --- | 1140 | 7 |
| *Nandinia binotata* | [[29](#_ENREF_29)] | KJ619487 | --- | --- | --- | --- | --- | --- | --- | 15393 | 100 |
| *Atilax paludinosus* | [[30](#_ENREF_30), [31](#_ENREF_31)] | --- | AF522325 | --- | AY974021 | --- | --- | --- | --- | 2183 | 14 |
| *Bdeogale nigripes* | [[31](#_ENREF_31)] | --- | AY950655 | --- | AY974022 | --- | --- | --- | --- | 2150 | 14 |
| *Bdeogale crassicauda* | [[31](#_ENREF_31)] | --- | AY950654 | --- | --- | --- | --- | --- | --- | 1137 | 7 |
| *Crossarchus platycephalus* |  | --- | KF670757 | --- | --- | --- | --- | --- | --- | 1140 | 7 |
| *Crossarchus alexandri* | [[30](#_ENREF_30), [31](#_ENREF_31)] | --- | AF522326 | --- | AY974024 | --- | --- | --- | --- | 2184 | 14 |
| *Crossarchus obscurus* | [[7](#_ENREF_7)] | --- | AY170101 | --- | AY170041 | --- | --- | --- | --- | 2184 | 14 |
| *Cynictis penicillata* | [[7](#_ENREF_7), [19](#_ENREF_19)] | --- | AF511060 | --- | AY170041 | --- | --- | --- | --- | 2184 | 14 |
| *Galerella pulverulenta* | [[30](#_ENREF_30), [31](#_ENREF_31)] | --- | AF522330 | --- | AY974026 | --- | --- | --- | --- | 2182 | 14 |
| *Galerella sanguinea* | [[31](#_ENREF_31), [32](#_ENREF_32)] | --- | JX630983 | --- | AY974027 | --- | --- | --- | --- | 2163 | 14 |
| *Galerella nigrata* | [[32](#_ENREF_32)] | --- | JX402114 | --- | --- | --- | --- | --- | --- | 1089 | 7 |
| *Helogale hirtula* | [[30](#_ENREF_30)] | --- | AF522335 | --- | --- | --- | --- | --- | --- | 1097 | 7 |
| *Helogale parvula* | [[30](#_ENREF_30), [31](#_ENREF_31)] | --- | AF522333 | --- | AY974028 | --- | --- | --- | --- | 2171 | 14 |
| *Herpestes naso* | [[30](#_ENREF_30), [31](#_ENREF_31)] | --- | AF522339 | --- | AY974032 | --- | --- | --- | --- | 2171 | 14 |
| *Herpestes javanicus* |  | AY873843 | --- | --- | --- | --- | --- | --- | --- | 15391 | 100 |
| *Herpestes edwardsii* | [[7](#_ENREF_7), [31](#_ENREF_31)] | --- | AY170107 | --- | AY974029 | --- | --- | --- | --- | 1202 | 8 |
| *Herpestes ichneumon* | [[30](#_ENREF_30), [31](#_ENREF_31)] | --- | AF522337 | --- | AY974030 | --- | --- | --- | --- | 2157 | 14 |
| *Herpestes brachyurus* | [[33](#_ENREF_33)] | --- | FJ391225 | --- | FJ391229 | --- | --- | --- | --- | 1202 | 8 |
| *Herpestes fuscus* | [[33](#_ENREF_33)] | --- | FJ391215 | --- | FJ391231 | --- | --- | --- | --- | 2175 | 14 |
| *Herpestes urva* | [[31](#_ENREF_31), [34](#_ENREF_34)] | --- | DQ519074 | --- | AY974033 | --- | --- | --- | --- | 2183 | 14 |
| *Ichneumia albicauda* | [[30](#_ENREF_30), [31](#_ENREF_31)] | --- | AF522341 | --- | AY974034 | --- | --- | --- | --- | 1202 | 8 |
| *Liberiictis kuhni* | [[22](#_ENREF_22), [31](#_ENREF_31)] | --- | AF522342 | --- | AY974035 | --- | --- | --- | --- | 2176 | 14 |
| *Mungos mungo* | [[7](#_ENREF_7)] | --- | AY170095 | --- | AY170035 | --- | --- | --- | --- | 2184 | 14 |
| *Rhynchogale melleri* | [[30](#_ENREF_30), [31](#_ENREF_31)] | --- | AF522344 | --- | AY974036 | --- | --- | --- | --- | 2107 | 14 |
| *Suricata suricatta* | [[7](#_ENREF_7)] | --- | AY170111 | --- | AY170054 | --- | --- | --- | --- | 2184 | 14 |
| *Crocuta crocuta* | [[35](#_ENREF_35)] | JF894379 | --- | --- | --- | --- | --- | --- | --- | 15395 | 100 |
| *Hyaena hyaena* | [[35](#_ENREF_35)] | JF894376 | --- | --- | --- | --- | --- | --- | --- | 15391 | 100 |
| *Hyaena brunnea* | [[36](#_ENREF_36)] | --- | AY928677 | --- | --- | --- | --- | --- | --- | 1140 | 7 |
| *Proteles cristatus* | [[36](#_ENREF_36)] | --- | AY928679 | --- | --- | --- | --- | --- | --- | 1140 | 7 |
| *Canis lupus* | [[37](#_ENREF_37)] | AB499825 | --- | --- | --- | --- | --- | --- | --- | 15379 | 100 |
| *Vulpes lagopus* | [[38](#_ENREF_38)] | KP200876 | --- | --- | --- | --- | --- | --- | --- | 15311 | 99 |
| *Martes flavigula* | [[39](#_ENREF_39)] | KM347744 | --- | --- | --- | --- | --- | --- | --- | 15307 | 99 |
| *Hippopotamus amphibius* | [[40](#_ENREF_40)] | NC_000889 | --- | --- | --- | --- | --- | --- | --- |  |  |
| *Steno bredanensis* | [[41](#_ENREF_41)] | JF339982 | --- | --- | --- | --- | --- | --- | --- |  |  |
| *Balaenoptera physalus* | [[42](#_ENREF_42)] | NC_001321 | --- | --- | --- | --- | --- | --- | --- |  |  |
| *Tapirus indicus* | [[43](#_ENREF_43)] | NC_023838 | --- | --- | --- | --- | --- | --- | --- |  |  |
| *Bos grunniens* |  | KR011113 | --- | --- | --- | --- | --- | --- | --- |  |  |
| *Equus caballus* | [[44](#_ENREF_44)] | NC_001640 | --- | --- | --- | --- | --- | --- | --- |  |  |
| *Erignathus barbatus* | [[45](#_ENREF_45)] | AM181027 | --- | --- | --- | --- | --- | --- | --- |  |  |
| *Procyon lotor* | [[46](#_ENREF_46)] | AB291073 | --- | --- | --- | --- | --- | --- | --- |  |  |
| *Tremarctos ornatus* | [[47](#_ENREF_47)] | FM177764 | --- | --- | --- | --- | --- | --- | --- |  |  |

# represent sequence only list in the published paper.

# References

1. Burger PA, Steinborn R, Walzer C, Petit T, Mueller M, Schwarzenberger F. Analysis of the mitochondrial genome of cheetahs (Acinonyx jubatus) with neurodegenerative disease. Gene. 2004;338(1):111-9. doi: 10.1016/j.gene.2004.05.020.

2. Johnson WE, Eizirik E, Pecon-Slattery J, Murphy WJ, Antunes A, Teeling E, et al. The Late Miocene radiation of modern Felidae: A genetic assessment. Science. 2006;311(5757):73-7. doi: DOI 10.1126/science.1122277.

3. Huang KH, Deng JB, Yu JQ, Cai ZG, Liu YL, Peng R. Complete mitochondrial genome sequence of the Asian golden cat, Catopuma temminckii. Mitochondr Dna. 2015:1-2. doi: 10.3109/19401736.2015.1007292.

4. Lopez JV, Cevario S, O'Brien SJ. Complete nucleotide sequences of the domestic cat (Felis catus) mitochondrial genome and a transposed mtDNA tandem repeat (Numt) in the nuclear genome. Genomics. 1996;33(2):229-46. doi: 10.1006/geno.1996.0188

5. Paijmans JL, Fickel J, Courtiol A, Hofreiter M, Forster DW. Impact of enrichment conditions on cross-species capture of fresh and degraded DNA. Mol Ecol Resour. 2015. doi: 10.1111/1755-0998.12420.

6. Fernandes CA, Ginja C, Pereira I, Tenreiro R, Bruford MW, Santos-Reis M. Species-specific mitochondrial DNA markers for identification of non-invasive samples from sympatric carnivores in the Iberian Peninsula. Conserv Genet. 2008;9(3):681-90. doi: 10.1007/s10592-007-9364-5.

7. Yoder AD, Burns MM, Zehr S, Delefosse T, Veron G, Goodman SM, et al. Single origin of Malagasy Carnivora from an African ancestor. Cah Rev The. 2003;421(6924):734-7. doi: 10.1038/nature01303.

8. Delisle I, Strobeck C. A phylogeny of the Caniformia (order Carnivora) based on 12 complete protein-coding mitochondrial genes. Mol Phylogenet Evol. 2005;37(1):192-201. doi: 10.1016/j.ympev.2005.04.025.

9. Tamada T, Siriaroonrat B, Subramaniam V, Hamachi M, Lin L-K, Oshida T, et al. Molecular diversity and phylogeography of the Asian leopard cat, Felis bengalensis, inferred from mitochondrial and Y-chromosomal DNA sequences. Zool Sci. 2008;25(2):154-63. doi: 10.2108/zsj.25.154.

10. Wu X, Zheng T, Jiang Z, Wei L. The mitochondrial genome structure of the clouded leopard (Neofelis nebulosa). Genome. 2007;50(2):252-7. doi: 10.1139/g06-143.

11. Wei L, Wu X, Zhu L, Jiang Z. Mitogenomic analysis of the genus Panthera. Science China Life Sciences. 2011;54(10):917-30. doi: 10.1007/s11427-011-4219-1.

12. Sun Y, Lu T, Sun Z, Guan W, Liu Z, Teng L, et al. Complete mitochondrial genome of a wild Siberian tiger. Mitochondr Dna. 2014. Epub 2014/03/26. doi: 10.3109/19401736.2013.840597.

13. Wei L, Wu X, Jiang Z. The complete mitochondrial genome structure of snow leopard Panthera uncia. Mol Biol Rep. 2009;36(5):871-8.

14. Veron G, Patou ML, Tóth M, Goonatilake M, Jennings AP. How many species of Paradoxurus civets are there? New insights from India and Sri Lanka. J Zool Syst Evol Res. 2014. doi: 10.1111/jzs.12085.

15. Patou ML, Debruyne R, Jennings AP, Zubaid A, Rovie-Ryan JJ, Veron G. Phylogenetic relationships of the Asian palm civets (Hemigalinae & Paradoxurinae, Viverridae, Carnivora). Mol Phylogenet Evol. 2008;47(3):883-92. doi: 10.1016/j.ympev.2008.03.026.

16. Veron G, Heard S. Molecular systematics of the Asiatic Viverridae (Carnivora) inferred from mitochondrial cytochrome b sequence analysis. J Zool Syst Evol Res. 2000;38(4):209-17. doi: 10.1046/j.1439-0469.2000.384132.x.

17. Wilting A, Fickel J. Phylogenetic relationship of two threatened endemic viverrids from the Sunda Islands, Hose's civet and Sulawesi civet. J Zool. 2012;288(3):184-90. doi: 10.1111/j.1469-7998.2012.00939.x.

18. Masuda R, Kaneko Y, Siriaroonrat B, Subramaniam V, Hamachi M. Genetic variations of the masked palm civet Paguma larvata, inferred from mitochondrial cytochrome b sequences. Mammal Study. 2008;33(1):19-24. doi: http://dx.doi.org/10.3106/1348-6160(2008)33[19:GVOTMP]2.0.CO;2.

19. Gaubert P, Tranier M, Delmas AS, Colyn M, Veron G. First molecular evidence for reassessing phylogenetic affinities between genets (Genetta) and the enigmatic genet‐like taxa Osbornictis, Poiana and Prionodon (Carnivora, Viverridae). Zool Scr. 2004;33(2):117-29. doi: 10.1111/j.1463-6409.2004.00140.x.

20. Hassanin A, Veron G. The complete mitochondrial genome of the Spotted Linsang, Prionodon pardicolor, the first representative from the family Prionodontidae (Mammalia, Carnivora). Mitochondr Dna. 2014;(0):1-2. doi: 10.3109/19401736.2014.926482.

21. Gaubert P, Begg CM. Re-assessed molecular phylogeny and evolutionary scenario within genets (Carnivora, Viverridae, Genettinae). Mol Phylogenet Evol. 2007;44(2):920-7. doi: 10.1016/j.ympev.2006.12.011.

22. Gaubert P, Fernandes CA, Bruford MW, Veron G. Genets (Carnivora, Viverridae) in Africa: an evolutionary synthesis based on cytochrome b sequences and morphological characters. Biol J Linn Soc. 2004;81(4):589-610. doi: 10.1111/j.1095-8312.2004.00309.x.

23. Gaubert P, Taylor PJ, Fernandes CA, Bruford MW, Veron G. Patterns of cryptic hybridization revealed using an integrative approach: a case study on genets (Carnivora, Viverridae, Genetta spp.) from the southern African subregion. Biol J Linn Soc. 2005;86(1):11-33. doi: 10.1111/j.1095-8312.2005.00518.x.

24. Gaubert P, Cordeiro-Estrela P. Phylogenetic systematics and tempo of evolution of the Viverrinae (Mammalia, Carnivora, Viverridae) within feliformians: implications for faunal exchanges between Asia and Africa. Mol Phylogenet Evol. 2006;41(2):266-78. doi: 10.1016/j.ympev.2006.05.034.

25. Hassanin A. The complete mitochondrial genome of the Servaline Genet, Genetta servalina, the first representative from the family Viverridae (Mammalia, Carnivora). Mitochondr Dna. 2014;(0):1-2. doi: 10.3109/19401736.2014.926479.

26. Weng HM, Wang L, Chan FT, Sun PY, Li KY, Ju YT. The complete mitochondrial genome of the small Indian civet, Viverricula indica taivana - the first complete representation of the genus Viverricula. Mitochondr Dna. 2014:1-2. doi: 10.3109/19401736.2014.958712.

27. Flynn JJ, Finarelli JA, Zehr S, Hsu J, Nedbal MA. Molecular phylogeny of the carnivora (mammalia): assessing the impact of increased sampling on resolving enigmatic relationships. Syst Biol. 2005;54(2):317-37. doi: 10.1080/10635150590923326.

28. van Vuuren BJ, Woolaver L, Goodman SM. Genetic population structure in the boky-boky (Carnivora: Eupleridae), a conservation flagship species in the dry deciduous forests of central western Madagascar. Anim Conserv. 2012;15(2):164-73. doi: DOI 10.1111/j.1469-1795.2011.00498.x.

29. Hassanin A. The complete mitochondrial genome of the African palm civet, Nandinia binotata, the only representative of the family Nandiniidae (Mammalia, Carnivora). Mitochondr Dna. 2014;(0):1-2. doi: 10.3109/19401736.2014.926478.

30. Veron G, Colyn M, Dunham AE, Taylor P, Gaubert P. Molecular systematics and origin of sociality in mongooses (Herpestidae, Carnivora). Mol Phylogenet Evol. 2004;30(3):582-98. doi: Doi 10.1016/S1055-7903(03)00229-X.

31. Perez M, Li B, Tillier A, Cruaud A, Veron G. Systematic relationships of the bushy-tailed and black-footed mongooses (genus Bdeogale, Herpestidae, Carnivora) based on molecular, chromosomal and morphological evidence. J Zool Syst Evol Res. 2006;44(3):251-9. doi: DOI 10.1111/j.1439-0469.2006.00359.x.

32. Rapson SA, Goldizen AW, Seddon JM. Species boundaries and possible hybridization between the black mongoose (Galerella nigrata) and the slender mongoose (Galerella sanguinea). Mol Phylogenet Evol. 2012;65(3):831-9. doi: 10.1016/j.ympev.2012.08.005.

33. Patou ML, McLenachan PA, Morley CG, Couloux A, Jennings AP, Veron G. Molecular phylogeny of the Herpestidae (Mammalia, Carnivora) with a special emphasis on the Asian Herpestes. Mol Phylogenet Evol. 2009;53(1):69-80. doi: 10.1016/j.ympev.2009.05.038.

34. Veron G, Patou ML, Pothet G, Simberloff D, Jennings AP. Systematic status and biogeography of the Javan and small Indian mongooses (Herpestidae, Carnivora). Zool Scr. 2007;36(1):1-10. doi: DOI 10.1111/j.1463-6409.2006.00261.x.

35. Bon C, Berthonaud V, Maksud F, Labadie K, Poulain J, Artiguenave F, et al. Coprolites as a source of information on the genome and diet of the cave hyena. P R Soc B. 2012;279(1739):2825-30. doi: DOI 10.1098/rspb.2012.0358.

36. Koepfli K-P, Jenks SM, Eizirik E, Zahirpour T, Van Valkenburgh B, Wayne RK. Molecular systematics of the Hyaenidae: relationships of a relictual lineage resolved by a molecular supermatrix. Mol Phylogenet Evol. 2006;38(3):603-20. doi: 10.1016/j.ympev.2005.10.017.

37. Matsumura S, Inoshima Y, Ishiguro N. Reconstructing the colonization history of lost wolf lineages by the analysis of the mitochondrial genome. Mol Phylogenet Evol. 2014;80:105-12. doi: 10.1016/j.ympev.2014.08.004.

38. Yan S-Q, Guo P-C, Yue Y, Li W-H, Bai C-Y, Li Y-M, et al. The complete sequence of the mitochondrial genome of Arctic fox (Alopex lagopus). Mitochondr Dna. 2015;(0):1-2. doi: 10.3109/19401736.2014.1003860.

39. Jang KH, Hwang UW. Complete mitochondrial genome of Korean yellow-throated marten, Martes flavigula (Carnivora, Mustelidae). Mitochondr Dna. 2014;(0):1-2.

40. Ursing BM, Arnason U. Analyses of mitochondrial genomes strongly support a hippopotamus-whale clade. Proceedings Biological sciences / The Royal Society. 1998;265(1412):2251-5. doi: 10.1098/rspb.1998.0567.

41. Vilstrup JT, Ho SY, Foote AD, Morin PA, Kreb D, Krutzen M, et al. Mitogenomic phylogenetic analyses of the Delphinidae with an emphasis on the Globicephalinae. Bmc Evol Biol. 2011;11:65. doi: 10.1186/1471-2148-11-65.

42. Valverde JR, Marco R, Garesse R. A conserved heptamer motif for ribosomal RNA transcription termination in animal mitochondria. Proc Natl Acad Sci U S A. 1994;91(12):5368-71.

43. Muangkram Y, Wajjwalku W, Kaolim N, Buddhakosai W, Kamolnorranath S, Siriaroonrat B, et al. The complete mitochondrial genome of the Asian tapirs (Tapirus indicus): the only extant Tapiridae species in the old world. Mitochondr Dna. 2016;27(1):413-5. doi: 10.3109/19401736.2014.898283.

44. Xu X, Arnason U. The complete mitochondrial DNA sequence of the horse, Equus caballus: extensive heteroplasmy of the control region. Gene. 1994;148(2):357-62. doi: 10.1016/0378-1119(94)90713-7.

45. Arnason U, Gullberg A, Janke A, Kullberg M, Lehman N, Petrov EA, et al. Pinniped phylogeny and a new hypothesis for their origin and dispersal. Mol Phylogenet Evol. 2006;41(2):345-54. doi: 10.1016/j.ympev.2006.05.022

46. Yonezawa T, Nikaido M, Kohno N, Fukumoto Y, Okada N, Hasegawa M. Molecular phylogenetic study on the origin and evolution of Mustelidae. Gene. 2007;396(1):1-12. doi: 10.1016/j.gene.2006.12.040.

47. Krause J, Unger T, Nocon A, Malaspinas AS, Kolokotronis SO, Stiller M, et al. Mitochondrial genomes reveal an explosive radiation of extinct and extant bears near the Miocene-Pliocene boundary. Bmc Evol Biol. 2008;8:220. doi: 10.1186/1471-2148-8-220.
