# Supplementary material for: Comprehensive species set revealing the phylogeny and biogeography of Feliformia (Mammalia, Carnivora) based on mitochondrial DNA
Source: PLoS One. 2017 Mar 30;12(3):e0174902. doi: 10.1371/journal.pone.0174902 (PMC5373635; doi:10.1371/journal.pone.0174902)
Supplement: S2 Table — (DOCX) [file pone.0174902.s003.docx]

| **S2 Table. Substitution models selected in PartitionFinder using the Bayesian information criterion (BIC).** | | |
| --- | --- | --- |
| No. of partitions | Partition | Model |
| 1 | *ATP6*_codon 1, *Cytb*_codon 1, *ND1*_codon 1, *ND4L*_codon 1, *ND6*_codon 3,t*RNA-PHE*, *tRNA-Ala*, *tRNA-Arg*, *tRNA-Asp*, *tRNA-Cys*, *tRNA*-*Gln*, *tRNA*-*Glu*, *tRNA*-*Gly*, *tRNA*-*His*, *tRNA*-*Ile*, *tRNA*-*Leu*, *tRNA*-*Leu2*, *tRNA*-*Lys*, *tRNA*-*Pro*, *tRNA*-*Ser*, *tRNA*-*Ser2*, *tRNA*-*Tyr* | GTR+I+G |
| 2 | *12S*, *16S*, *ATP8*_codon 1, *ATP8*_codon 2, *ND2*_codon 1, *ND3*_codon 1, *ND4*_codon 1, *ND5*_codon 1, *tRNA*-*Asn*, *tRNA*-*Thr*, *tRNA*-*Trp*, *tRNA*-*Val* | GTR+I+G |
| 3 | *ATP6*_codon 2, *COX1*_codon 2, *COX2*_codon 2, *COX3*_codon 2, *Cytb*_codon 2, *ND1*_codon 2, *ND2*_codon 2, *ND3*_codon 2, *ND4L*_codon 2, *ND4*_codon 2, *ND5*_codon 2, *tRNA*-*Met* | GTR+I+G |
| 4 | *Cytb*_codon 3, *ND1*_codon 3, *ND2*_codon 3 | GTR+I+G |
| 5 | *COX1*_codon 1, *COX2*_codon 1, *COX3*_codon 1, *ND6*_codon 2 | GTR+I+G |
| 6 | *COX1_*codon3 | GTR+I+G |
| 7 | *ATP6*_codon 3, *ATP8*_codon 3, *COX2*_codon 3, *COX3*_codon 3, *ND3*_codon 3, *ND4L*_codon3, *ND4*_codon 3, *ND5*_codon 3, *ND6*_codon 1 | GTR+I+G |
