# Supplementary material for: Comprehensive species set revealing the phylogeny and biogeography of Feliformia (Mammalia, Carnivora) based on mitochondrial DNA
Source: PLoS One. 2017 Mar 30;12(3):e0174902. doi: 10.1371/journal.pone.0174902 (PMC5373635; doi:10.1371/journal.pone.0174902)
Supplement: S3 Table — (DOCX) [file pone.0174902.s004.docx]

| **S3 Table. Uncorrected p-distance between Cyt b fragments from 7 families within Feliformia** | | | | | | | |
| --- | --- | --- | --- | --- | --- | --- | --- |
|  | (1) | (2) | (3) | (4) | (5) | (6) | (7) |
| (1) Felidae |  |  |  |  |  |  |  |
| (2) Eupleridae | 0.166 |  |  |  |  |  |  |
| (3) Herpestidae | 0.180 | 0.164 |  |  |  |  |  |
| (4) Hyaenidae | 0.187 | 0.166 | 0.177 |  |  |  |  |
| (5) Viverridae | 0.176 | 0.171 | 0.172 | 0.185 |  |  |  |
| (6) Nandiniidae | 0.150 | 0.152 | 0.160 | 0.170 | 0.161 |  |  |
| (7)Prionodontidae | 0.189 | 0.180 | 0.184 | 0.191 | 0.181 | 0.177 |  |
